# Thematic Selection: Stress and Stress-related Disorders Neurobiology & Translational Aspects of Stress-related Disorders (Part 3)

**DOI:** 10.2174/1570159X2205231107115145

**Published:** 2023-11-07

**Authors:** Agorastos Agorastos

**Affiliations:** 1 Assistant Professor of Psychiatry, Aristotle University of Thessaloniki, Greece

   Optimal neuroendocrine coordination of the stress system and its reactivity is essential to survival, proper development, health, and well-being. Acute and chronic stress exposure can disrupt optimal neuroendocrine reactivity and has been implicated as a major downstream pathway of biological, emotional, and behavioural vulnerability, thus, mediating a repeatedly well-documented higher risk for mental and physical (co-)morbidity [1, 2]. However, there is still little recognition of the importance of stress-system neuroendocrinology within most medical disciplines, and only a few stress-system-related implications flow into research and clinical practice. Novel approaches are needed for the proper understanding, neuroendocrine assessment, and efficacious management of stress system dysregulation in both mental and physical stress-related disorders, especially in view of the particular challenges of the evolving new lifestyle of modern societies [3].

   This current issue (Part 3) of the special thematic selection entitled “Stress and Stress-related Disorders” handles a comprehensive collection of nine articles on neurobiological and translational aspects of stress research.

   In two clinically relevant reviews, Su *et al.* provided an interesting overview of prospective clinical trials on promising novel anti-inflammatory strategies for the treatment of major depressive disorder (MDD) [4], while Menke reviewed promising study results of MDD treatment alternatives targeting the hypothalamic-pituitary-adrenal axis, such as corticotrophin-releasing hormone 1, vasopressin V1B and glucocorticoid receptor antagonists as well as FKBP5 antagonists [5].

   Another five articles focus on molecular pathophysiological pathways in the development of stress-related disorders. In an exceptional review, Ell *et al*. provide available animal and human findings on epigenetics with respect to fear, anxiety, and stress-related states with an emphasis on histone modifications [6]. Kyriatzis *et al.* review current findings on the involvement of the neurotensinergic system in stress response and the pathophysiology of stress-related disorders, with an emphasis on gene, mRNA, and protein alterations, behavioral and pharmacological studies and therapeutic implementations [7]. Providing insights from mass spectrometry and nuclear magnetic resonance metabolomic studies in rodent models, Papageorgiou *et al.* investigate affected molecular pathways upon stress exposure and provide insightful information on stress-induced systemic changes in the brain and the periphery [8]. The review article by MacKay *et al.* focuses on the role of the gut-immune-brain axis, the human microbiome and the therapeutic potential of pro and prebiotics in stress-related disorders [9]. Finally, Lochner *et al.* conducted a systematic review on post-mortem brain tissue studies to provide an overview of quantitative and qualitative studies on DNA methylation, cellular and molecular alterations, and gene expression profiling in brain areas associated with OCD [10].

   The last two articles have focused on stress-related functional and structural imaging studies. Paltoglou *et al.* offer an overview of functional MRI and functional connectivity analysis studies, which support the coordination of the stress response by three main dynamic networks [11]. On the other hand, Cardoner *et al.* provide an overview of structural imaging studies investigating the impact of stress on brain morphology [12].

   All these articles provide most intriguing insights in stress-related biological mechanisms. Further understanding of pathways susceptible to disruption following acute and chronic stress exposure could help us better identify the pathophysiologic trajectories that link stress to multilevel biological changes but also develop new translational diagnostic approaches as well as novel, neurobiology-driven treatments.
